# Optimization of Culture Medium Enhances Viable Biomass Production and Biocontrol Efficacy of the Antagonistic Yeast, *Candida diversa*

**DOI:** 10.3389/fmicb.2017.02021

**Published:** 2017-10-17

**Authors:** Jia Liu, Guangkun Li, Yuan Sui

**Affiliations:** ^1^Chongqing Key Laboratory of Economic Plant Biotechnology, Collaborative Innovation Centre of Special Plant Industry in Chongqing, College of Forestry and Life Science, Institute of Special Plants, Chongqing University of Arts and Sciences, Chongqing, China; ^2^School of Food Science and Engineering, Hefei University of Technology, Hefei, China

**Keywords:** antagonistic yeast, antioxidant system, biocontrol activity, biomass, medium optimization

## Abstract

Viable biomass production is a key determinant of suitability of antagonistic yeasts as potential biocontrol agents. This study investigated the effects of three metal ions (magnesium, ferrous, and zinc) on biomass production and viability of the antagonistic yeast, *Candida diversa*. Using response surface methodology to optimize medium components, a maximum biomass was obtained, when the collective Mg^2+^, Fe^2+^, and Zn^2+^ concentrations were adjusted in a minimal mineral (MM) medium. Compared with the unmodified MM, and three ion-deficient MM media, yeast cells cultured in the three ion-modified MM medium exhibited a lower level of cellular oxidative damage, and a higher level of antioxidant enzyme activity. A biocontrol assay indicated that *C. diversa* grown in the ion-modified MM exhibited the greatest level of control of gray mold on apple fruit. These results provide new information on culture medium optimization to grow yeast antagonists in order to improve biomass production and biocontrol efficacy.

## Introduction

A variety of fungal pathogens cause post-harvest diseases on fruits, vegetables and grains, which result in significant economic losses. Some post-harvest diseases also represent a potential health risk to humans, as certain decay fungi produce mycotoxins (Yang et al., 2015; Liu et al., 2017). Currently, synthetic fungicides are still the main method used to manage post-harvest decay. Increasing concerns about environmental and food safety have generated a great interest in the development of alternative control methods (Mari et al., 2014). Biological control, utilizing antagonistic yeasts, has been proposed as an effective and eco-friendly alternative. Research on this approach has been actively pursued over the past 30 years, and a few yeast-based biocontrol products are commercially available (Liu et al., 2013a; Sui et al., 2015; Spadaro and Droby, 2016; Wisniewski et al., 2016). Several species of yeast in the genus *Candida*, including *C. diversa* (Li et al., 2016), *C. sake* (Marín et al., 2016), *C. oleophila* (Liu et al., 2013b) and *C. saitoana* (El Ghaouth et al., 2003), have been reported as effective post-harvest biocontrol agents.

Commercial production of biocontrol agents need to produce sufficient biomass in an economic manner. In addition, formulated products must have adequate shelf life, while retaining biocontrol efficacy (Liu et al., 2009; Melin et al., 2011). The ability to achieve these goals requires low-cost medium components that produce a maximum level of viable biomass. In this regard, metal ions in a medium are required to sustain certain biochemical reactions important in the growth and viability of yeast. Thus, they play a critical role in biomass production and maintaining viability (Poreda et al., 2013). Magnesium, ferrous, and zinc ions, can significantly impact enzyme activity, lipid synthesis, biomass accumulation, and viability (Jernejc and Legiša, 2002). For example, Stehlik-Tomas et al. (2004) reported that the addition of zinc, copper, and manganese sulfate to a molasses-based medium enhanced the biomass yield of *Saccharomyces cerevisae* up to 30% under semi-aerobic conditions.

The current study evaluated the ability of the addition of metal ions to a minimal mineral medium (MM) to maximize biomass production of the antagonistic yeast, *C. diversa*. The study investigated the effect of Mg^2+^, Fe^2+^, and Zn^2+^ ions on (i) viable biomass production; (ii) amelioration of oxidative damage to proteins and lipids, and the enhancement of antioxidant enzyme activity; and (iii) the biocontrol efficacy of *C. diversa* against gray mold on apple fruit caused by *Botrytis cinerea*.

## Materials and Methods

### Fungal Pathogen

The fungal pathogen, *B. cinerea*, was isolated from infected apple fruit and maintained on potato dextrose agar (PDA). To reactivate the culture and verify its pathogenicity, the pathogen was inoculated into wounded apple fruit and re-isolated onto PDA once an infection was established. *B. cinerea* spore suspension was obtained from 2-week-old PDA cultured at 25°C. Spore concentration was determined using a hemocytometer and adjusted to 10^4^ spores/mL with sterile distilled water.

### Fruit Host

Apple fruits (*Malus* x *domestica* Borkh. cv. Fuji) were harvested at commercial maturity. Fruits without wounds or rot were selected based on uniformity of size. The selected fruits were disinfected with 2% (v/v) sodium hypochlorite for 2 min, rinsed with tap water, and air-dried prior to their use in the biocontrol assays.

### Yeast Strain and Growth Conditions

The yeast, *C. diversa* L-198, was isolated from the surface of plum fruit (Li et al., 2016). *C. diversa* was cultured at 25°C for 48 h on yeast peptone dextrose agar (YPDA, 10 g of yeast extract, 20 g of peptone, 20 g of dextrose, and 2% agar in 1 L of water). A flask culture (20 mL in 100-mL conical flask) of a single colony was carried out in sterilized liquid YPD medium overnight as a seed culture. The seed culture was centrifuged and washed twice using sterile water, and then transferred to a MM media (Fan et al., 2013) at an initial concentration of 3 × 10^6^ cells/mL. On a per liter basis, the MM was composed of: 0.5 g MgSO_4_⋅7H_2_O, 3.0 mg FeSO_4_⋅7H_2_O, 4.5 mg ZnSO_4_⋅7H_2_O, 5.0 g (NH_4_)SO_4_, 3.5 g KH_2_PO_4_, 15.0 mg EDTA, 4.5 mg CaCl_2_⋅2H_2_O, 1.0 mg H_3_BO_3_, 0.4 mg Na_2_MoO_4_⋅2H_2_O, 0.3 mg CoCl_2_⋅2H_2_O, 0.3 mg CuSO_4_⋅5H_2_O, 0.1 mg KI, 50 ug D-biotin, 0.2 mg *p*-aminobenzoic acid, 1.0 mg nicotinic acid, 1.0 mg calcium pantothenate, 1.0 mg pyridoxine HCl, 1.0 mg thiamine HCl, and 25.0 mg myoinositol. Glucose (20 g/L) was added as a carbon source. All media were adjusted to pH 5.0 before seed cultures were added. The yeast cultures were grown in 200 ml in a 1-L conical flask shaken at 200 rpm and kept at 28°C.

### Response Surface Methodology (RSM) for Optimizing Medium Components

Five initial concentrations of Mg^2+^ (0, 500, 1000, 1500, and 2000 mg/L), Fe^2+^ (0, 1.2, 2.2, 3.2, and 4.2 mg/L), and Zn^2+^ (0, 3, 26.5, 50, and 73.5 mg/L) were evaluated in single factor experiments, in order to determine maximum biomass production of the yeast after 96-h of culture. The concentrations of Mg^2+^, Fe^2+^, and Zn^2+^ gaining each maximum biomass served as non-code variables of A, B, and C, respectively. The code variables of X_1_, X_2_, and X_3_ were transformed from non-code variables as follows, X_1_ = (A-1500)/500, X_2_ = (B-2.2)/1, X_3_ = (C-26.5)/23.5 (**Table 1**). Based on the data of the single factor experiments, a three-factor (Mg^2+^, Fe^2+^, and Zn^2+^) Box–Behnken design with 17 experimental runs was generated using DesignExpert^TM^ V.10 software (Hallenbeck et al., 2015). A quadratic polynomial equation was fitted to data in order to correlate the relationship between the independent variables and responses. RSM provided the ability to predict the optimum concentration of metal ions needed to provide maximum biomass production of the yeast.

**Table 1:** Levels of the code values and none code values based on single factor (ion) experiments.

Independent variables	Code	None code	Code levels (mg/L)		
			-1	0	1
Mg^2+^	X_1_	A	1000	1500	2000
Fe^2+^	X_2_	B	1.2	2.2	3.2
Zn^2+^	X_3_	C	3.0	26.5	50.0

*The relations between the code values and none code values were as follows. X_*1*_ = (A-1500)/500, X_*2*_ = (B-2.2)/1, X_*3*_ = (C-26.5)/23.5*.

### Analysis of Biomass and Viability

The unmodified MM, and the Mg^2+^, Fe^2+^, and Zn^2+^-deficient MM media were compared to the previously determined optimized medium. Yeast samples, cultured in the media described above, were collected after 96-h of culture, pelleted at 8,000 *g* for 3 min, and washed twice with sterile distilled water to remove residual medium. The samples were then divided two groups to determine dry biomass and cell viability, respectively.

Biomass was assessed as dry weight (g) per liter of culture medium (g/L). Specifically, yeast samples from 1 L of culture medium were oven-dried at 60°C until a stable weight was achieved. Yeast viability was determined using the methylene blue technique (Alfenore et al., 2002). A 200 μL sterile solution of methylene blue (0.3 mM in 68 mM Na_3_ citrate) was mixed with 200 μL of a yeast suspension and then diluted to reach an OD_620_ nm of 0.4–0.7. The mixture was shaken and, after 5 min of incubation, placed in a cell counting chamber. The number of stained and unstained yeast cells was separately counted in five different microscopic fields achieving a total of at least 200–300 counted stained and unstained cells. The percentage of viable cells was calculated as the number of unstained cells (live cells) divided by the total number of cells (stained and unstained cells). Measurements were made on three replicates of yeast grown in the different types of medium, and the experiment was repeated three times.

### Determination of Protein Carbonylation and Lipid Peroxidation

Carbonyl content and malondialdehyde (MDA) content were used as indicators of oxidative damage to proteins and lipids, respectively (Chi et al., 2015; Cheng et al., 2016). For the assay of carbonyl content, yeast samples were pulverized in liquid nitrogen. Proteins were extracted from the samples using 500 μL of 50 mM KH_2_PO_4_ buffer (pH 7.5) containing 10 mM Tris, 2 mM MgCl_2_, 2 mM EGTA, and 1 mM phenylmethylsulfonyl fluoride. Aliquots of extract were then reacted with 500 μL of 10 mM 2,4-dinitrophenylhydrazine (DNPH) dissolved in 2.5 M HCl or 2.5 M HCl without DNPH (blank control) in the dark at room temperature with vortexing of the reaction mixture every 15 min for 1 h. Proteins were precipitated with 20% (w/v) trichloroacetic acid (TCA) for 10 min on ice. After centrifugation at 3000 *g* for 20 min, protein pellets were washed with ethanol-ethyl acetate (1:1, v/v) and dissolved in 6 M guanidine hydrochloride with 20 mM KH_2_PO_4_ (pH 2.3). Absorbance was recorded at 380 nm after centrifugation at 9,500 *g* for 10 min. The carbonyl content was calculated using the molar absorption coefficient of 22,000/M/cm and expressed as nmol per mg protein.

A method based on the reaction of thiobarbituric acid with MDA was used for assaying lipid peroxidation, and detection of thiobarbituric acid-reactive species was determined. Pulverized yeast samples were resuspended in 500 μL of 50 mM KH_2_PO_4_ buffer (pH 6.0) containing 10% (w/v) TCA and centrifuged at 3,000 *g* for 10 min. Supernatants were mixed with 100 μL of 0.1 M EDTA and 600 μL of 1% (w/v) thiobarbituric acid. The reaction mixture was incubated at 100°C for 15 min and subsequently placed on ice for 10 min. After cooling down, absorbance was monitored at 532 nm. The MDA content was calculated using the molar absorption coefficient of 153,000/M/cm and expressed as nmol per mg protein (Chi et al., 2015; Cheng et al., 2016). Protein content was measured using the Bradford assay (Bradford, 1976), with bovine serum albumin as a standard. Measurements were made on three replicates of yeast grown in the different types of medium, and the experiment was repeated three times.

### Assay of Enzyme Activity

The extracts prepared from *C. diversa* that were used to determine the antioxidant enzyme activity of glutathione peroxidase (GPX) and superoxide dismutase (SOD) were prepared as previously described (Chi et al., 2015; Cheng et al., 2016), with slight modification. Yeast samples were pulverized in liquid nitrogen and the ground yeast cells were then suspended in chilled potassium phosphate buffer (0.1 M, pH 7.0). The cell homogenate from each sample was centrifuged at 10,000 *g* for 20 min at 4°C, and the supernatant was used for enzyme assay. The enzyme activity of GPX and SOD was assayed using commercial assay kits purchased from Nanjing Jiancheng Bioengineering Institute (Nanjing, China), and expressed as U per mg protein. One unit of GPX activity was defined as the oxidation of 1 μmol/L reduced glutathione to the oxidized glutathione per minute in the reaction system (Cheng et al., 2016). One unit of SOD activity was defined as the amount of enzyme causing 50% inhibition in the reduction rate of nitroblue tetrazolium (NBT) (Chi et al., 2015). Protein content was measured using the Bradford assay (Bradford, 1976). Measurements were made on three replicates of yeast grown in the different types of medium.

### Biocontrol Assay

Biocontrol efficacy of *C. diversa* was evaluated as described in a previous study (Li et al., 2016). Three wounds (4 mm deep × 3 mm wide) were made on the equator of each fruit. A 5-μL suspension (1 × 10^7^ cells/mL) of *C. diversa* cells was then pipetted to each wound. Fruits were allowed to air dry for 2 h and then a 5-μL spore suspension of *B. cinerea* (1 × 10^4^ spores/mL) was pipetted into each wound. Inoculation with sterile distilled water (no yeast), plus *B. cinerea* spores, served as a control. Treated fruits were placed in a covered plastic food tray, and each tray was enclosed in a polyethylene bag and stored at 25°C with an approximate 90% relative humidity (RH) in a programmable environmental chamber. Disease incidence and lesion diameter of apple fruits were determined after 4 days. Each treatment contained three replicates with 20 fruits per replicate, and the experiment was repeated three times.

## Results and Discussion

### Optimization of Metal Ions in the Medium

**FIGURE 1:**
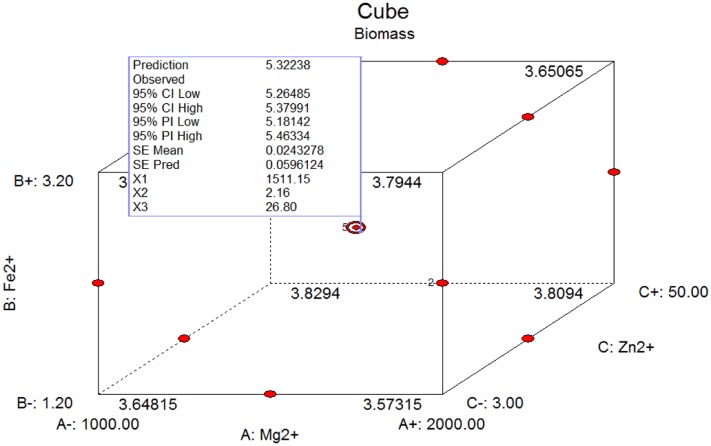
The integrative effect of metal ions (Mg^2+^, Fe^2+^, and Zn^2+^) on biomass based on RSM analysis.

The presence and concentration of metal ions, including magnesium, ferrous and zinc ions, affect microbial biomass production and their microbial metabolism (Kośmider et al., 2012; Hallenbeck et al., 2015; Abd Elrazak et al., 2017). The biomass of *C. diversa* was evaluated in single factor (ion) experiments. Maximum biomass was obtained in each single factor when the concentrations of Mg^2+^, Fe^2+^, and Zn^2+^ were 1500, 2.2, and 26.5 mg/L, respectively. Box–Behnken experimental design was then used to generate 17 experimental runs varying the concentration of the different metal ions, and the response values were then evaluated (**Table 2**). The quadratic polynomial equation was generated: Y = 5.32+0.03X_1_-0.04X_2_+0.01X_3_+0.05X_1_X_2_+0.01X_1_X_3_-0.1X_2_X_3_-0.63X_1_^2^-0.46X_2_^2^-0.55X_3_^2^ (*R*^2^ = 0.995). The Model *F*-value of 163.92 indicated that the model was significant, and was confirmed by an ANOVA analysis (Supplementary Table S1). The maximum dry biomass predicted by RSM was 5.32 g/L, when 1511.15 mg/L Mg^2+^, 2.16 mg/L Fe^2+^, and 26.80 mg/L Zn^2+^ were adjusted in the MM medium (**Figure 1**). Based on the optimization of the medium components, the effect of the optimized medium on the biomass production and viability of *C. diversa* was determined. The unmodified MM and the combined Mg^2+^, Fe^2+^, and Zn^2+^-deficient MM media served as comparisons.

**Table 2:** Box–Behnken experimental design matrix employed by DesignExpert^TM^ V.10 with experimental results to predict optimum concentrations of medium components.

Run	Design matrix			Dry biomass (g/L)
	A	B	C	
1	1000	3.2	26.5	4.16
2	1500	1.2	3.0	4.26
3	1000	2.2	50.0	4.07
4	2000	1.2	26.5	4.19
5	2000	2.2	50.0	4.21
6	2000	3.2	26.5	4.27
7	1500	1.2	50.0	4.49
8	2000	2.2	3.0	4.18
9	1500	2.2	26.5	5.33
10	1500	2.2	26.5	5.27
11	1500	3.2	3.0	4.33
12	1500	2.2	26.5	5.39
13	1000	1.2	26.5	4.30
14	1000	2.2	3.0	4.09
15	1500	2.2	26.5	5.29
16	1500	3.2	50.0	4.17
17	1500	2.2	26.5	5.33

### Effect of Optimized Concentrations of Metal Ions on Biomass Production and Viability

**FIGURE 2:**
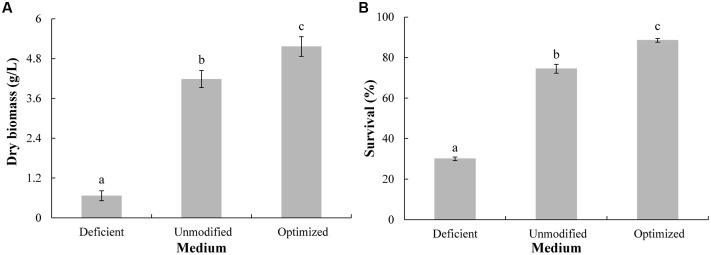
Biomass production (g DW/L) **(A)** and viability **(B)** of *Candida diversa* after 96-h culture in three different media: unmodified minimal mineral (MM) medium, ion-deficient MM medium, and Mg^2+^, Fe^2+^, and Zn^2+^ optimized MM medium. Data represent the mean ± SD of the pooled data from three experiments (*n* = 9). Columns with different letters indicate significant differences according to a Duncan’s multiple range test (*P* < 0.05).

Relative to the ion-deficient MM and the unmodified MM, the optimized MM produced the greatest dry biomass (**Figure 2A**), as well as the highest viability level after the 96-h cultivation (**Figure 2B**). These data indicate that the maximum viable biomass could be obtained in the optimized MM medium. Moreover, the dry biomass obtained in the optimized MM was 5.21 ± 0.30 g/L, thus confirming the reliability of determining the composition of the optimized medium by RSM.

### Protein Carbonylation and Lipid Peroxidation

**FIGURE 3:**
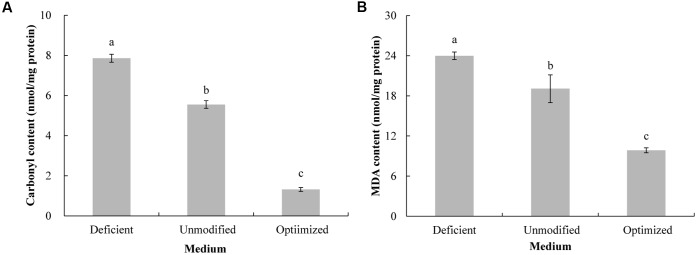
Protein carbonylation **(A)** and lipid peroxidation **(B)** in yeast cells of *C. diversa* after 96-h culture in three different media: unmodified MM medium, ion-deficient MM medium, and Mg^2+^, Fe^2+^, and Zn^2+^ optimized MM medium. Data represent the mean ± SD of the pooled data from three experiments (*n* = 9). Columns with different letters indicate significant differences according to a Duncan’s multiple range test (*P* < 0.05).

A certain level of protein and lipid oxidation occur in yeast cells during culturing which has a deleterious effect on the structure and function of proteins and lipids, and a concomitant decrease in viability (Liu et al., 2012; Johansson et al., 2016). Carbonyl content of proteins can be used as an indicator of the level of oxidative damage to proteins (Abegg et al., 2010), while MDA content can serve as an indicator of lipid peroxidation (Li et al., 2010). Comparing the three different types of media, the lowest carbonyl (**Figure 3A**) and MDA (**Figure 3B**) levels after 96-h cultivation were observed in yeast cultured in the optimized MM, while the highest levels were present in yeast grown in the ion-deficient MM. These data corresponded well with the results on biomass production and viability of *C. diversa* grown in the three different MM.

### Antioxidant Enzyme Activity

**FIGURE 4:**
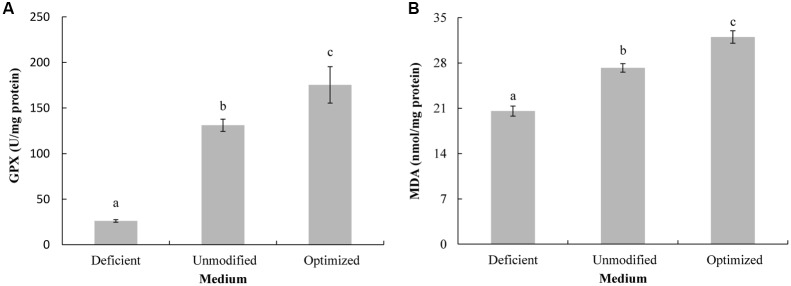
GPX **(A)** and SOD **(B)** enzyme activity in yeast cells of *C. diversa* after 96-h culture in three different media: unmodified MM medium, ion-deficient MM medium, and Mg^2+^, Fe^2+^, and Zn^2+^ optimized MM medium. Data represent the mean ± SD of the pooled data from three experiments (*n* = 9). Columns with different letters indicate significant differences according to a Duncan’s multiple range test (*P* < 0.05).

Antioxidant enzyme activity (GPX and SOD) was assessed in order to determine if the lower level of oxidative injury to proteins and lipids was associated with an increase in GPX and SOD activity, due to higher concentration of metal ions in the optimized medium. Amelioration of oxidative damage is partially dependent on the ability of antioxidant enzymes, such as GPX and SOD, to detoxify reactive oxygen species (ROS). These enzymes have been shown to positively contribute to stress adaptation in cultured yeast cells (Liu et al., 2011; Ribeiro et al., 2015; Cheng et al., 2016). In the present study, both GPX (**Figure 4A**) and SOD (**Figure 4B**) activity in *C. diversa* grown in optimized MM, was greater than in yeast grown in the other two types of MM (ion-deficient MM and non-modified MM). Antioxidant enzyme activity was lowest in yeast grown in the ion-deficient MM. These data suggest that the optimization of the concentration of metal ions (Mg^2+^, Fe^2+^, and Zn^2+^) enhances antioxidant enzyme activity, thus ameliorating the oxidative damage to proteins and lipids that normally occurs during culturing. Similar results were reported by Wiseman (2005), who indicated that Fe^2+^ and Zn^2+^ enhanced SOD and GPX activity in *Saccharomyces cerevisiae,* and reduced oxidative stress.

### Biocontrol Assay

**FIGURE 5:**
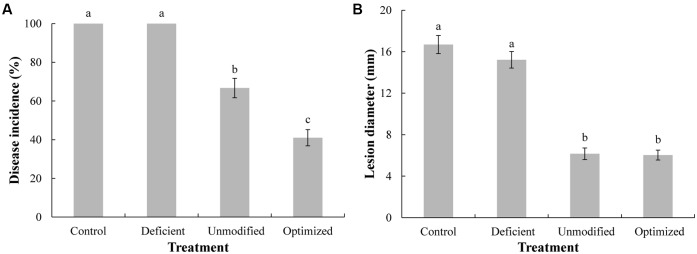
Biocontrol efficacy of *C. diversa* against *Botrytis cinerea* on apple fruit. Prior to use, yeast were grown for 96-h in three different media: unmodified minimal media (MM), ion-deficient MM, and Mg^2+^, Fe^2+^, and Zn^2+^ optimized MM. Control: sterile water plus *B. cinerea* spores, without *C. diversa*. Disease incidence **(A)** and lesion diameter **(B)** in apple fruits were measured 4 days after inoculation. Data represent the mean ± SD of the pooled data from three experiments (*n* = 9). Columns with different letters indicate significant differences according to a Duncan’s multiple range test (*P* < 0.05).

High levels of viability are advantageous for antagonistic yeasts used as biocontrol agent, where competition for nutrients and space plays a major role in biocontrol activity (Liu et al., 2013a; Wisniewski et al., 2016). As indicated in **Figure 5A**, the lowest level (percentage) of disease incidence of gray mold on apple fruit was observed in the treatment group utilizing *C. diversa* that had been cultured in optimized MM, followed by yeast grown in unmodified MM. The highest levels of disease incidence were observed with yeast grown in ion-deficient MM, whose level was not significantly different than the non-yeast, control group. Similar results were obtained for lesion diameter (**Figure 5B**), indicating that *C. diversa* affected both spore germination and germ tube or hyphal development. Notably, *C. diversa* grown in the ion-deficient MM had no impact on either disease incidence or lesion diameter, relative to the control. This result may have been due to the reduced level of viability in yeast grown in the ion-deficient MM (**Figure 2B**). These results confirm a previous study on other biocontrol yeasts. In this regard, Liu et al. (2009) reported that the antagonistic yeasts, *Cryptococcus laurentii* and *Pichia membranaefaciens*, with higher viability after liquid culture, exhibited better biocontrol performance.

## Conclusion

The present study provides information on the enhancement in biomass production, viability, and biocontrol efficacy of the antagonistic yeast, *C. diversa*, by optimization of the composition of the culture medium. Optimized concentrations of Mg^2+^, Fe^2+^, and Zn^2+^ enhanced the activity of antioxidant enzymes (GPX and SOD) and thus significantly reduced the level of oxidative injury that occurs to yeast cells during culture. The optimized medium allowed for higher biomass production, increased viability, and increased biocontrol efficacy. These results have practical implications for the large-scale production of biocontrol agents.

## Author Contributions

YS conceived and designed the experiments. JL and GL performed the experiments. JL analyzed the data. JL and YS drafted the manuscript. All authors read and approved the final manuscript.

## Conflict of Interest Statement

The authors declare that the research was conducted in the absence of any commercial or financial relationships that could be construed as a potential conflict of interest.
