# Supplementary material for: Optimization of Culture Medium Enhances Viable Biomass Production and Biocontrol Efficacy of the Antagonistic Yeast, Candida diversa
Source: Front Microbiol. 2017 Oct 17;8:2021. doi: 10.3389/fmicb.2017.02021 (PMC5650984; doi:10.3389/fmicb.2017.02021)
Supplement: Supplementary file 1 [file Table_1.DOCX]

**Table S1**

ANOVA for the response surface quadratic model.

| Source | Coefficients of regression equation | SS | df | MS | *F*-value | *P*-value |
| --- | --- | --- | --- | --- | --- | --- |
| Model | – | 4.37 | 9 | 0.49 | 163.92 | < 0.0001 |
| Intercept | 5.32 | – | – | – | – | – |
| A-Mg | 0.03 | 0.01 | 1 | 0.01 | 2.43 | 0.1629 |
| B-Fe | -0.04 | 0.01 | 1 | 0.01 | 3.93 | 0.0880 |
| C-Zn | 0.01 | 0.00 | 1 | 0.00 | 0.24 | 0.6410 |
| AB | 0.05 | 0.01 | 1 | 0.01 | 3.90 | 0.0888 |
| AC | 0.01 | 0.00 | 1 | 0.00 | 0.26 | 0.6289 |
| BC | -0.10 | 0.04 | 1 | 0.04 | 12.19 | 0.0101 |
| A^2^ | -0.63 | 1.69 | 1 | 1.69 | 569.81 | < 0.0001 |
| B^2^ | -0.46 | 0.89 | 1 | 0.89 | 299.96 | < 0.0001 |
| C^2^ | -0.55 | 1.28 | 1 | 1.28 | 432.94 | < 0.0001 |
| Residual | – | 0.02 | 7 | 0.00 | – | – |
| Lack of fit | – | 0.0118938 | 3 | 0.003965 | 1.7941727 | 0.2876 |

Note: R^2^ = 0.995, Adj-R^2^ (adjusted) = 0.989. SS - sum of squares, df - degree of freedom, MS - mean square.
